# Supplementary material for: Introduction of Electron Donor Groups into the Azulene Structure: The Appearance of Intense Absorption and Emission in the Visible Region
Source: Molecules. 2024 Jul 17;29(14):3354. doi: 10.3390/molecules29143354 (PMC11279626; doi:10.3390/molecules29143354)
Supplement: Supplementary file 1 [file molecules-29-03354-s001.zip › molecules-3086375-supplementary.pdf]

Supplementary Materials

Contents

1. Spectra of compounds

2. Density Functional Theory (DFT) Calculations

3. Cyclic voltammetry studies

1.Spectra of compounds

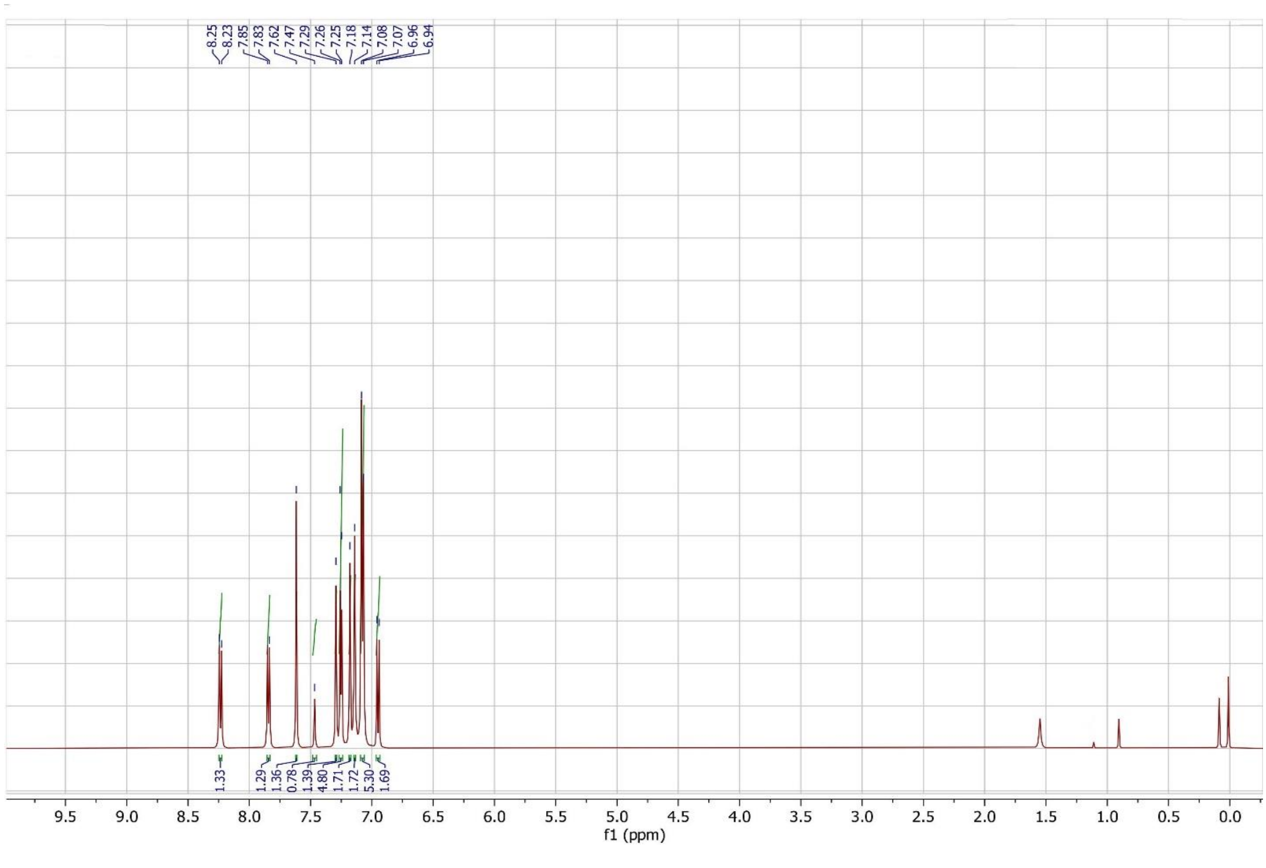

Figure S1. ^{1}H NMR spectra of 2-(N,N-diphenylaniline)-azulene **4**.

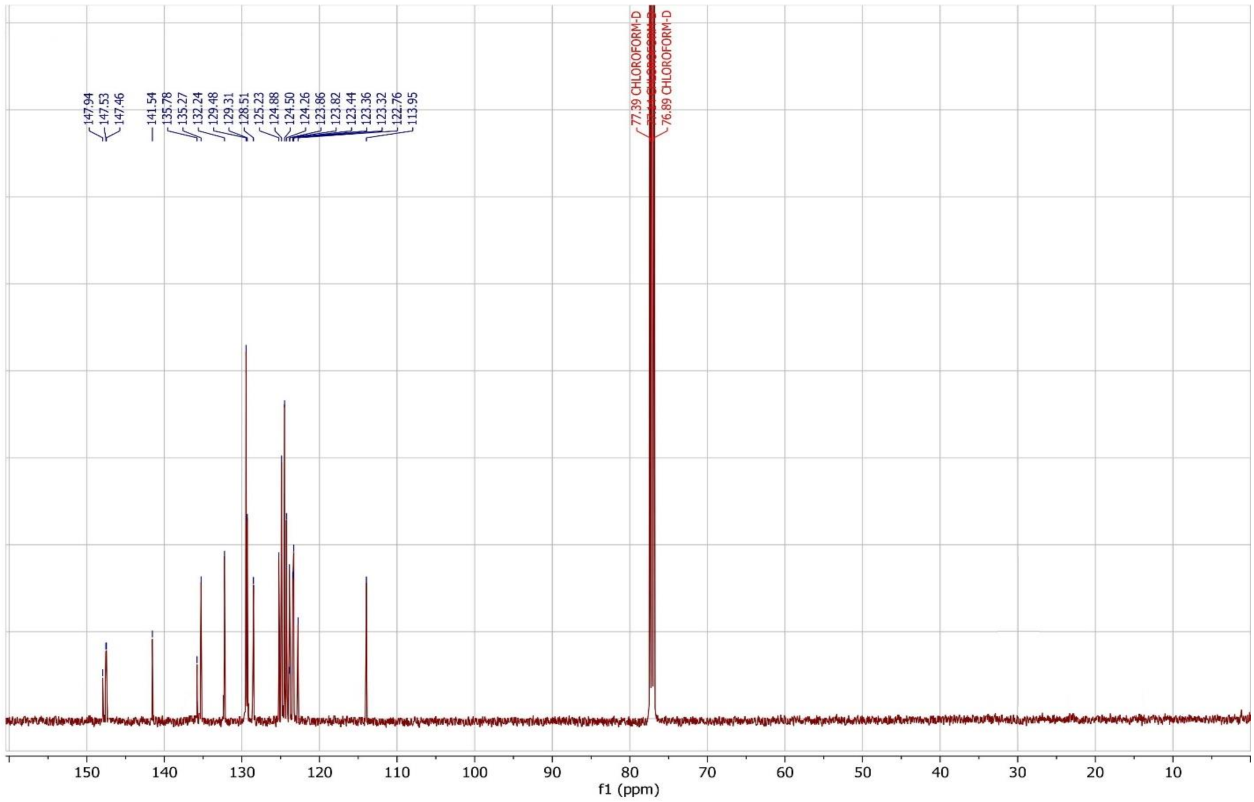

Figure S2. ^{13}C NMR spectra of 2-(N,N-diphenylaniline)-azulene **4**

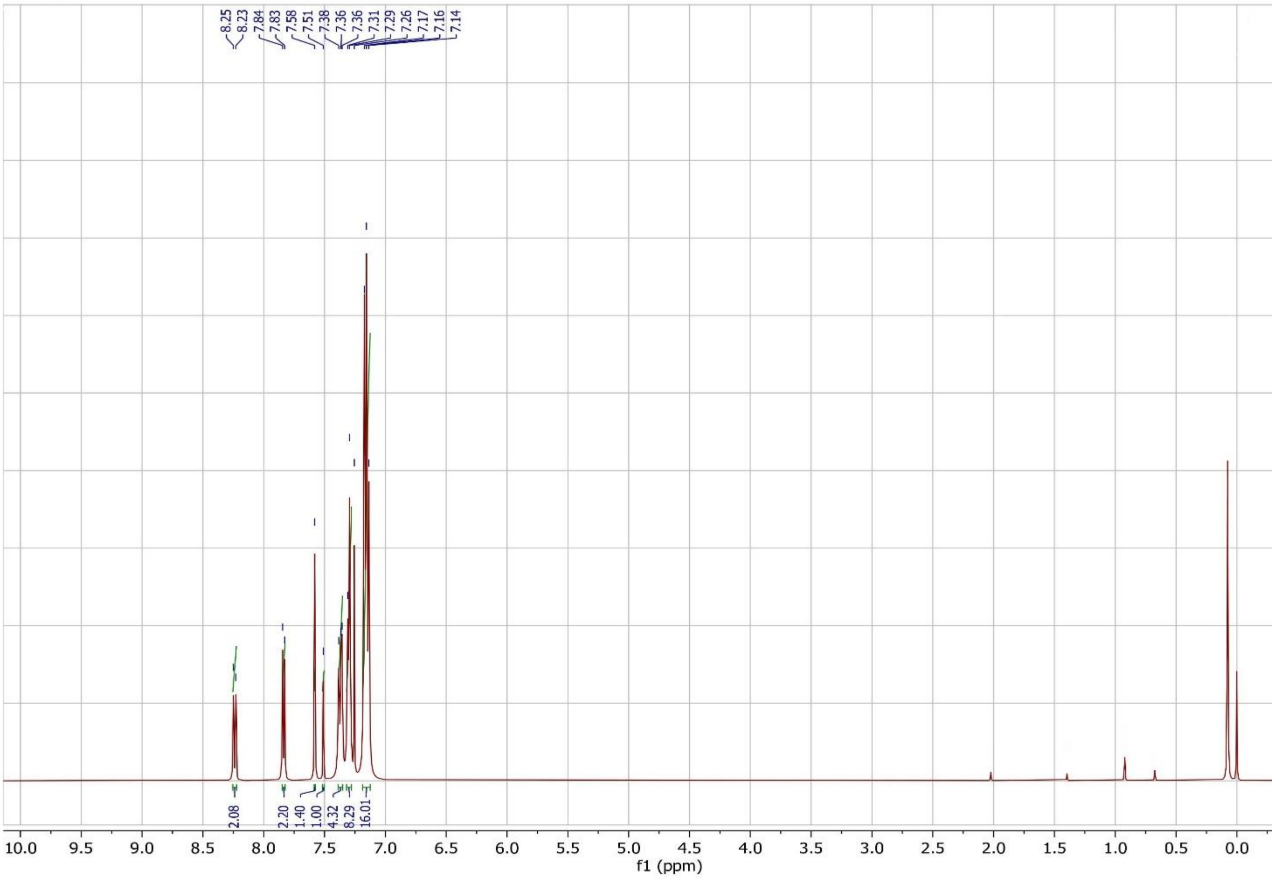

Figure S3. ^{1}H NMR spectra of 2,6-bis(N,N-diphenylaniline)-azulene **6**

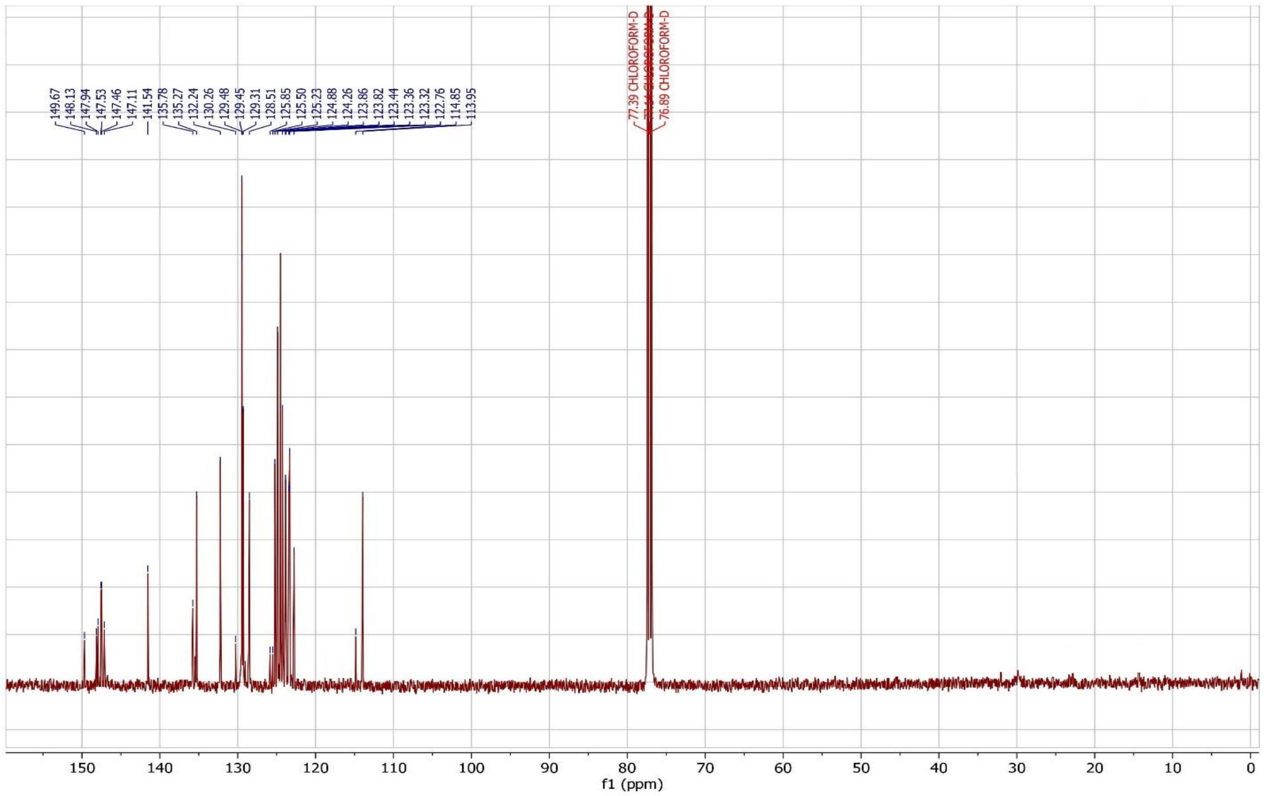

Figure S4. ^{13}C NMR spectra of 2,6-bis(N,N-diphenylaniline)-azulene **6**

2. Density Functional Theory (DFT) Calculations

To characterize the geometry and orbitals of HOMO-LUMO diphenylaniline-azulenes **4** and **6**, DFT calculations based on the level of B3LYP/6-31G * (d, p) Gaussian 16 were used. The geometry of the molecules was optimized in a singlet state using the keywords Opt and Freq, and to visualize the frontier orbitals, the results were recorded in a .chk. file.

After the correct completion of the calculations, the .chk file was opened in GaussView 6.0 and the HOMO-LUMO orbitals were visualized from it using the built-in GaussView tool. Then we obtained the energies of the frontier orbitals in Hartree units and recalculated them into eV, multiplying by the recalculation coefficient 27.2114.

Table S1. Atomic coordinates of optimized geometry of **4**

Center Number	Atomic Number	Atomic Type	Coordinates (Angstroms) X	Y	Z
1	6	0	-0.739023	1.022974	0.646219
2	6	0	0.652673	1.021641	0.637578
3	6	0	1.389620	-0.000062	0.000064
4	6	0	0.652661	-1.021740	-0.637451
5	6	0	-0.739046	-1.023058	-0.646085
6	6	0	-1.460948	-0.000051	0.000083
7	6	0	-3.605176	-1.231239	0.112019
8	6	0	-3.195993	-2.216984	1.029407
9	6	0	-3.899658	-3.419775	1.129401
10	6	0	-5.027858	-3.652721	0.333141
11	6	0	-5.442272	-2.669150	-0.573358

12	6	0	-4.735162	-1.469989	-0.691960
13	6	0	-5.442193	2.669297	0.573042
14	6	0	-4.735216	1.470079	0.691733
15	6	0	-3.605061	1.231279	-0.112011
16	6	0	-3.195569	2.217055	-1.029215
17	6	0	-3.899112	3.419921	-1.129292
18	6	0	-5.027481	3.652903	-0.333298
19	7	0	-2.884972	-0.000001	0.000069
20	6	0	7.506089	0.855973	0.935746
21	6	0	8.102589	0.000007	-0.000084
22	6	0	7.506062	-0.855986	-0.935874
23	6	0	6.146473	-1.081897	-1.177655
24	6	0	5.035579	-0.511610	-0.550955
25	6	0	5.035596	0.511500	0.550983
26	6	0	6.146505	1.081829	1.177614
27	6	0	3.688858	-0.785681	-0.841659
28	6	0	2.855546	-0.000065	0.000057
29	6	0	3.688881	0.785502	0.841792
30	1	0	-1.275515	1.817243	1.152118
31	1	0	1.178419	1.831171	1.132101
32	1	0	1.178381	-1.831273	-1.131998
33	1	0	-1.275531	-1.817309	-1.152016
34	1	0	-2.331341	-2.034960	1.657559
35	1	0	-3.572186	-4.170752	1.841642
36	1	0	-5.575492	-4.585480	0.418179
37	1	0	-6.311534	-2.839334	-1.200982
38	1	0	-5.051019	-0.716259	-1.404309
39	1	0	-6.311589	2.839522	1.200470
40	1	0	-5.051289	0.716325	1.403961
41	1	0	-2.330775	2.035022	-1.657170
42	1	0	-3.571388	4.170914	-1.841399
43	1	0	-5.575026	4.585709	-0.418392
44	1	0	8.194126	1.423937	1.557441
45	1	0	9.191100	0.000029	-0.000121
46	1	0	8.194082	-1.423924	-1.557610
47	1	0	5.920003	-1.803983	-1.961325
48	1	0	5.920057	1.803901	1.961303
49	1	0	3.353541	-1.467371	-1.611150
50	1	0	3.353592	1.467171	1.611314

Method: DFT B3LYP 6-31G*
Key word: opt = freq
E (RB3LYP): -1134,087739 hartree

Table S2. Atomic coordinates of optimized geometry of **6**

Center Number	Atomic Number	Atomic Type	Coordinates (Angstroms) X	Y	Z
1	6	0	-1.845237	-1.136905	0.186932
2	6	0	-2.678851	-0.000294	-0.000389
3	6	0	-1.845185	1.136272	-0.187803
4	6	0	-0.499694	0.738334	-0.120370
5	6	0	0.619752	1.566933	-0.235901
6	6	0	1.977558	1.248545	-0.176471
7	6	0	2.616740	-0.000450	-0.000688
8	6	0	1.977510	-1.249415	0.175085
9	6	0	0.619685	-1.567730	0.234628
10	6	0	-0.499722	-0.739065	0.119244
11	7	0	-8.418154	0.000030	0.000399
12	6	0	-6.993593	-0.000053	0.000160
13	6	0	-6.272247	-1.075342	0.555407

14	6	0	-4.880542	-1.073426	0.547079
15	6	0	-4.143921	-0.000225	-0.000230
16	6	0	-4.880564	1.073060	-0.547343
17	6	0	-6.272275	1.075146	-0.555288
18	6	0	-9.136738	-1.213209	-0.238871
19	6	0	-8.721380	-2.101259	-1.248734
20	6	0	-9.423640	-3.287641	-1.474743
21	6	0	-10.556566	-3.600278	-0.713273
22	6	0	-10.976752	-2.713789	0.285960
23	6	0	-10.271161	-1.532959	0.530326
24	6	0	-9.136452	1.213486	0.239542
25	6	0	-8.720999	2.101339	1.249533
26	6	0	-9.422900	3.287950	1.475459
27	6	0	-10.555567	3.600992	0.713769
28	6	0	-10.975857	2.714687	-0.285585
29	6	0	-10.270610	1.533628	-0.529867
30	7	0	8.381235	0.000097	-0.000346
31	6	0	6.958192	-0.000116	-0.000458
32	6	0	6.236263	0.818280	-0.891049
33	6	0	4.843411	0.821166	-0.881041
34	6	0	4.108335	-0.000437	-0.000694
35	6	0	4.843447	-0.821921	0.879710
36	6	0	6.236312	-0.818739	0.889939
37	6	0	9.101448	-1.218293	0.211657
38	6	0	10.228381	-1.241131	1.053897
39	6	0	10.936391	-2.429788	1.248841
40	6	0	10.525523	-3.613341	0.623359
41	6	0	9.400184	-3.594035	-0.209876
42	6	0	8.695947	-2.406559	-0.423976
43	6	0	9.101317	1.218667	-0.211509
44	6	0	10.229607	1.241567	-1.051981
45	6	0	10.937626	2.430324	-1.246139
46	6	0	10.525485	3.613980	-0.621665
47	6	0	9.398847	3.594615	0.209787
48	6	0	8.694540	2.407030	0.423123
49	1	0	-2.181288	-2.155844	0.323014
50	1	0	-2.181191	2.155236	-0.323806
51	1	0	0.399546	2.623516	-0.385621
52	1	0	2.647666	2.099635	-0.256521
53	1	0	2.647573	-2.100549	0.255044
54	1	0	0.399437	-2.624302	0.384363
55	1	0	-6.809610	-1.904723	1.000738
56	1	0	-4.353172	-1.904049	1.003785
57	1	0	-4.353218	1.903618	-1.004194
58	1	0	-6.809642	1.904603	-1.000471
59	1	0	-7.853358	-1.856474	-1.850374
60	1	0	-9.091224	-3.962370	-2.257550
61	1	0	-11.103191	-4.519587	-0.895690
62	1	0	-11.849576	-2.946917	0.887974
63	1	0	-10.592029	-0.855722	1.313725
64	1	0	-7.853180	1.856200	1.851323
65	1	0	-9.090418	3.962536	2.258361
66	1	0	-11.101927	4.520472	0.896118
67	1	0	-11.848487	2.948140	-0.887753
68	1	0	-10.591571	0.856528	-1.313346
69	1	0	6.772102	1.439933	-1.598957
70	1	0	4.315951	1.438978	-1.600371
71	1	0	4.316032	-1.439865	1.598957
72	1	0	6.772145	-1.440317	1.597917
73	1	0	10.541656	-0.329416	1.549846
74	1	0	11.803647	-2.432373	1.901750
75	1	0	11.073931	-4.535930	0.782143
76	1	0	9.075967	-4.502914	-0.707142

77	1	0	7.834317	-2.392279	-1.081863
78	1	0	10.543895	0.329800	-1.547188
79	1	0	11.805914	2.432934	-1.897677
80	1	0	11.073923	4.536648	-0.779870
81	1	0	9.073598	4.503538	0.706298
82	1	0	7.831915	2.392814	1.079695

Method: DFT B3LYP 6-31G*
Key word: opt = freq
E (RB3LYP): -1882,418693 hartree

3. Cyclic voltammetry studies

Electrochemical studies were carried out in acetonitrile solvent containing tetrabutylammonium hexafluorophosphate ($^{n}Bu_4NPF_6$) under argon atmosphere. Ag/AgCl electrode, glassy carbon electrodes and platinum electrodes were used as reference electrodes for working electrodes and counter electrodes, respectively. 50 μL of an ethanol solution of compound **4** (or **6**) at a concentration of 5 mg/mL was pipetted and dripped onto a glass-carbon electrode, which was evaporated under pressure from a wash flask at room temperature (the circular opening of the electrode was 4 mm in diameter).
